# Supplementary material for: Impact of retreatment with an artemisinin-based combination on malaria incidence and its potential selection of resistant strains: study protocol for a randomized controlled clinical trial
Source: Trials. 2013 Sep 23;14:307. doi: 10.1186/1745-6215-14-307 (PMC3849445; doi:10.1186/1745-6215-14-307)
Supplement: Additional file 1 — Follow-up chart for the pre-randomized controlled trial phase. [file 1745-6215-14-307-S1.pdf]

Additional file 1 Follow-up Chart Pre-RCT Phase

Day	0	1	2	14[1]	28[1]	42[1]	Any other day[1]
History (symptoms)	X			X	X	X	X
Examination (clinical)	X	X	X	X*	X*	X	X
Temperature	X	X	X	X*	X*	X	X
Rapid diagnostic test	X						
Blood film	X		X*	X*	X*	X	X*
Filter paper PCR	X		X*	X*	X*	X	X*
Informed consent	X						
Haematology	X						
Serum sample	X						
Treatment	X	X	X				
Adverse events	X	X	X	X	X	X	X
Concomitant medications	X	X	X	X	X	X	X

X = perform this task; * only if symptoms indicate a possible clinical treatment failure

[1] For a clinical treatment failure see day 0 RCT phase
