# Supplementary material for: Impact of retreatment with an artemisinin-based combination on malaria incidence and its potential selection of resistant strains: study protocol for a randomized controlled clinical trial
Source: Trials. 2013 Sep 23;14:307. doi: 10.1186/1745-6215-14-307 (PMC3849445; doi:10.1186/1745-6215-14-307)
Supplement: Additional file 2 — Follow-up chart for the randomized controlled trial phase (and post-randomized controlled trial). [file 1745-6215-14-307-S2.pdf]

Additional file 2 Follow-up Chart RCT Phase (and post-RCT)

Day	0	1	2	3	4-6	7	14[1]	21[1]	28[1]	Any other day[1]
History (symptoms)	X					X	X	X	X	X
Examination (clinical)	X	X	X	X		X	X	X	X	X
Temperature	X	X	X	X		X	X	X	X	X
Blood film	X	X	X	X		X	X	X	X	X
Filter paper PCR	X	X	X	X		X	X	X	X	X
Informed consent	X									
Haematology	X						X		X	X
Serum sample	X									
Treatment	X	X	X	X[1]	X[1]					
Adverse events	X	X	X	X	X[1]	X	X	X	X	X
Concomitant medications	X	X	X	X	X[1]	X	X	X	X	X

X[1] = quinine + clindamycin treatment administration; only for RCT phase

[1] For a treatment failure see day 0 Post-RCT phase
